# Beyond the expected: left ventricular myocardial hydatid cyst a case report

**DOI:** 10.1093/omcr/omae141

**Published:** 2024-11-25

**Authors:** Safae Lanjeri, Kaoutar Imrani, Oumaima Mesbah, Nabil Mouatassim Billah, Ittimade Nassar

**Affiliations:** Department of Radiology, Ibn Sina University Hospital, Faculty of Medicine and Pharmacy of Rabat, Mohamed V University, Rabat, Morocco; Department of Radiology, Ibn Sina University Hospital, Faculty of Medicine and Pharmacy of Rabat, Mohamed V University, Rabat, Morocco; Department of Radiology, Ibn Sina University Hospital, Faculty of Medicine and Pharmacy of Rabat, Mohamed V University, Rabat, Morocco; Department of Radiology, Ibn Sina University Hospital, Faculty of Medicine and Pharmacy of Rabat, Mohamed V University, Rabat, Morocco; Department of Radiology, Ibn Sina University Hospital, Faculty of Medicine and Pharmacy of Rabat, Mohamed V University, Rabat, Morocco

**Keywords:** hydatid cyst, Echinococcus, cardiac, MRI

## Abstract

Hydatid disease is an infection caused by the larval form of Echinococcus. It is a zoonosis primarily affecting the lungs and liver. While pulmonary involvement is most common, the cysts can develop in various extrapulmonary sites within the thorax, such as the pleural cavity, fissures, mediastinum, heart, vascular structures, chest wall, and diaphragm. However, intracardiac localization of hydatid cyst is very rare and it is found in less than 2% of cases. Cardiac involvement can be caused by systemic or pulmonary circulation, or by direct spread from adjacent structures. Imaging techniques, particularly MRI, play a crucial role not only in diagnosing hydatid cysts, but also in assessing their extension and identifying complications. We report a case of a 13-year-old girl, with a left ventricular myocardial hydatid cyst.

## Introduction

Hydatid disease is an infection caused by Echinococcus granulosus larvae. It is still an endemic parasitic infection in many sheep-raising nations. Domestic dogs and cats are primary carriers of these organisms by consumption of infested and improperly disposed offal. Humans, acting as intermediary hosts, contract the infection by ingesting unwashed, uncooked vegetables containing the parasite’s eggs.

After ingestion of contaminated food, the larva migrates to the liver via the portal circulation and may disseminate to the lungs and other organ systems*.* Hydatidosis typically manifests in the liver and lungs. Cardiac involvement is rare, representing only 0.5%–2% of cases [1].

Detecting cardiac hydatid cysts in their early stages can be difficult due to the prolonged period between exposure to the parasite and symptom onset [1]. Although these cysts may not show symptoms initially, they can later manifest as chest pain, dyspnea, palpitations and ventricular tachycardia, fibrillation, obstruction of blood flow from the heart chambers and atrioventricular nodal blocks.

Different imaging modalities can be used to aid diagnosis. Magnetic Resonance Imaging (MRI) offers excellent soft tissue contrast, distinguishing circulating blood and soft tissue while providing a comprehensive view of cardiac anatomy [2]. However, experience with MRI findings in patients with cardiac hydatid disease remains limited. This article aims to describe MRI features of cardiac hydatid disease.

## Case report

**Figure 1:**
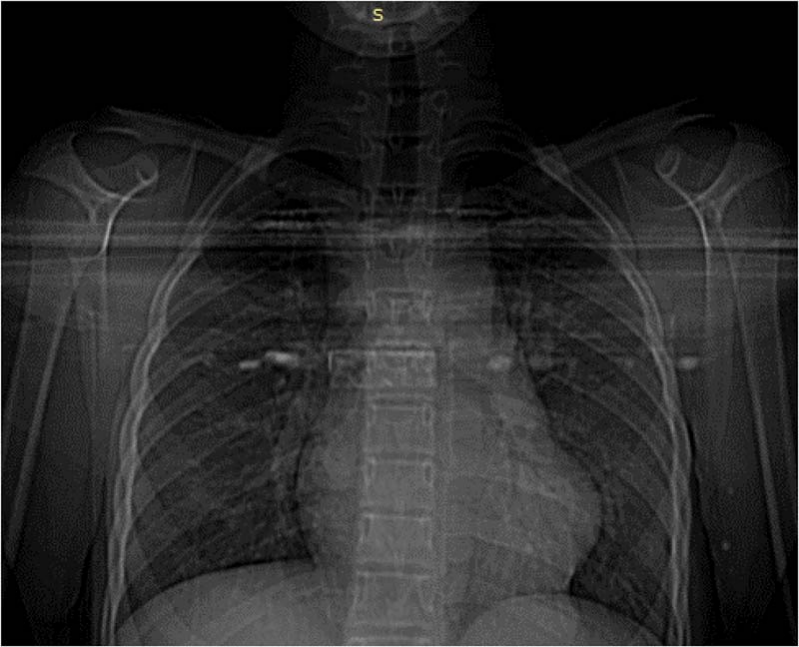
Posterior anterior chest XR: Left ventricle and heart dimensions are increased.

A 13-year-old girl, living in rural area, with no prior medical history, presented with a chief complaint of cough, shortness of breath and palpitations. A general examination showed stable vitals with a regular pulse, a heart rate of 74 bpm and normal blood pressure of 120/80 mm/Hg. Heart sounds were normal. Laboratory tests showed a raised eosinophil count. ECG was normal. Chest X-ray was performed and showed mild cardiomegaly (Fig. 1).

**Figure 2:**
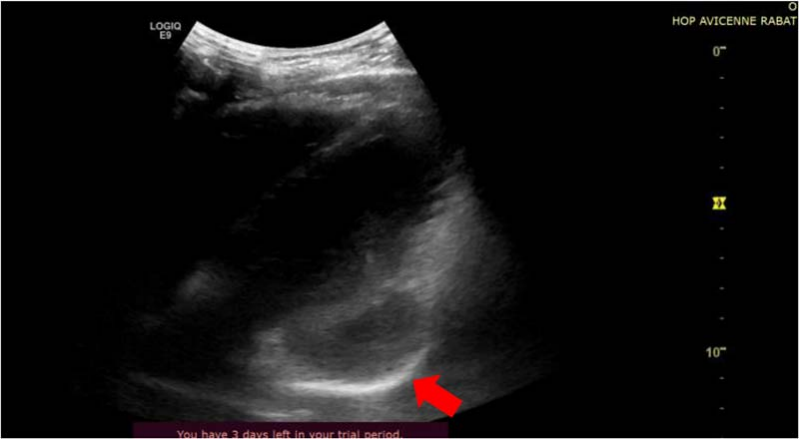
Trans-thoracic four chamber echocardiographic view showing a cystic mass (arrow) on the anterior lateral segment of the left ventricle.

Transthoracic echocardiography demonstrated a cystic lesion adjacent to the anterior-lateral segment of the left ventricle (Fig. 2). The left ventricle (LV) ejection fraction was 60%, the right ventricle (RV) size and function were normal.

She was referred to the department of radiology for a chest CT for further investigation.

**Figure 3:**
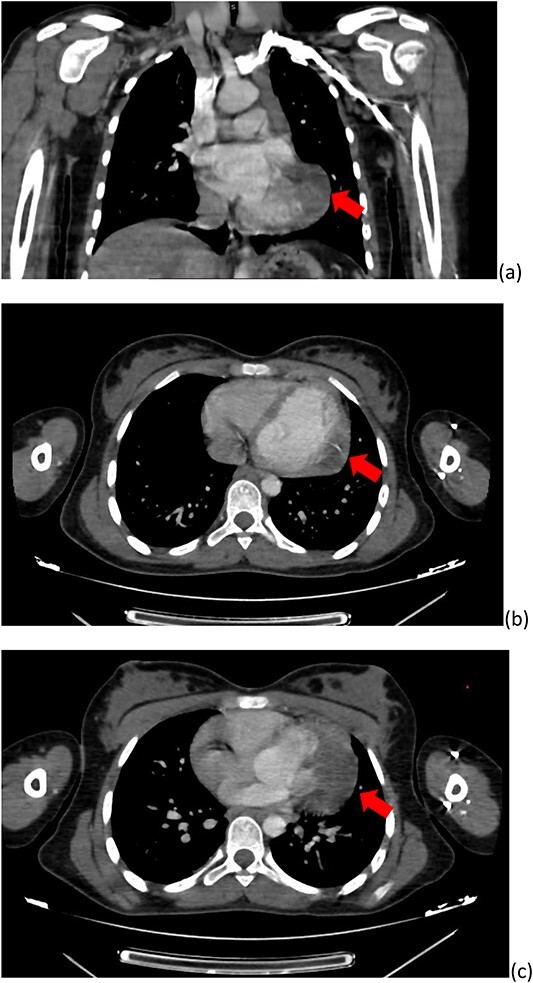
Axial (**a**) (**b**) and coronal (**c**) thoracic computed tomography with contrast revealing hypodense cystic lesion on left ventricular myocardium (arrow) not enhancing after contrast injection.

CT with contrast administration revealed a non-enhancing hypo-intense cystic lesion on the left ventricular wall. (B) Coronal and (C) axial-abdominal tomographic images reveal hypo-intense cystic lesions in the liver with approximately 10 HU density (Fig. 3).

**Figure 4:**
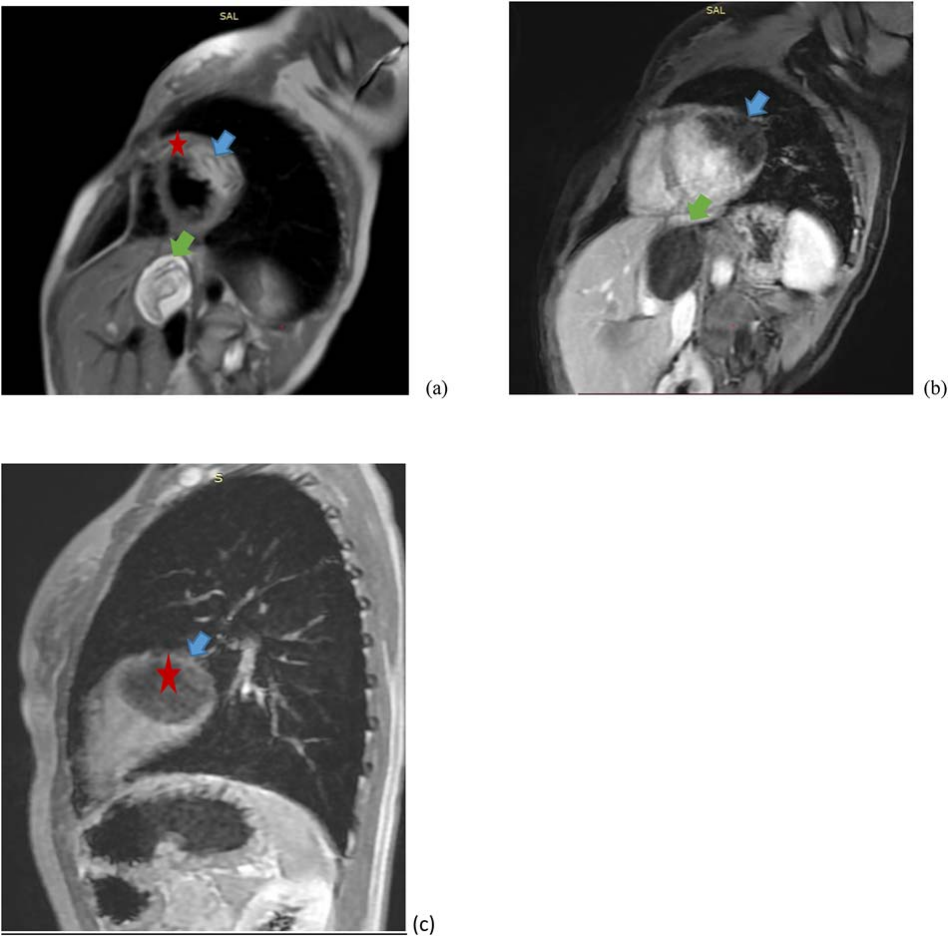
A 13 year-old femal patient presented with a chief complaint of cough, shortness of breath and palpitations. The cyst that includes membranes, is hyperintense on T2-weighted images (**A**) and mildly hypointense on T1-weighted axial (**B** and **C**) images (star) located in left ventricle posterior wall myocardium is.

A cardiac magnetic resonance imaging was advised for detailed evaluation, which showed a hyperintense T2 signal cystic lesion (blue arrow) with a peripheral hypointense rim, located in the myocardium of the left ventricle with hypo intense membranous structure in it (Fig 4).

In addition, sagittal T2-weighted images confirmed another cystic lesion in the liver (green arrow).

Albendazole 400 mg/PO every 12 h was started and cystectomy under extracorporeal circulation was done.

## Discussion

The first reported case of cardiac hydatid cyst was documented by Williams in 1936 [2].

The eggs of Echinococcus granulosus can disseminate to the heart through several pathways, including the coronary circulation, pulmonary veins, lymphatic vessels of the intestines, thoracic duct, superior and inferior vena cava, and even the hemorrhoidal veins of the large intestine.

The most common location in the heart is the left ventricle (60%), followed by the right ventricle (15%), the interventricular septum (9%), the left atrium (8%), the right atrium (4%), and interatrial septum (2%) respectively [3].

Transthoracic echocardiography serves as the primary imaging technique for cardiac evaluation [4, 5]. However, it has notable limitations, being operator dependent, with restricted field of view in individuals with a larger body size and a limited visualization of the left ventricular apex and right heart chambers. Computed tomography (CT) offers valuable insights by distinguishing cysts from solid tumors like myxomas or fibromas through CT density measurements and contrast enhancement analysis post intravenous injection [6], surpassing transthoracic echocardiography in specificity. However, CT imaging comes with drawbacks such as significant radiation exposure and lower temporal resolution compared to echocardiography and cardiac MRI [1].

Advancements in cardiac MRI technology, including increased magnet strengths, surface coil channels, post-processing techniques, and sophisticated myocardial soft-tissue characterization sequences, have transformed it into a powerful diagnostic tool for various complex cardiac conditions [7]. Magnetic resonance imaging is an excellent modality with higher soft tissue contrast that allow distinguishing circulating blood and soft tissue.

A cardiac hydatid cyst usually appears as an oval or spherical lesion that is hypointense on T1-weighted images and with a signal intensity more than or equal to cerebrospinal fluid on T2-weighted images. The presence of a hypointense peripheral ring on T2-weighted images, representing the pericyst, is often described as a characteristic feature of hydatid cysts in literature [6]. Walther et al. introduced a hypothesis associating the appearance of the hypointense peripheral ring on T2-weighted images with the developmental stages of hydatid cysts. According to their proposal, simple viable hydatid cysts could display a low-intensity rim surrounding the high-signal content within the cyst [1].

Similarly, MRI delineates the complications of cardiac hydatidosis comprehensively. Cysts protruding into the cardiac cavity indicate potential intracavitary rupture. Intracavitary cyst rupture is identifiable on bright blood SSFP gradient images by hypointense filling defects within the atrium or ventricle. Rupture of cardiac hydatid cysts into the pericardium results in pericarditis, evidenced by gadolinium-enhanced MRI showing pericardial thickening and enhancement [8]. A large Echinococcal cyst located in a ventricular cavity may obstruct valve leaflet coaptation, leading to valvular dysfunction. Phase-contrast MRI quantifies valve regurgitation accurately. MR imaging effectively demonstrates mechanical outflow obstruction by the cyst, both morphologically and functionally [8].

Cardiac surgery is the treatment of choice for most cases of cardiac hydatid cyst [9]. Complications associated with cardiac surgery are more common in patients with pre-existing conditions such as diabetes and renal failure. Preoperative agents are used with the aim of obtaining a softening of cyst wall and a reduction in intracystic pressure in order to facilitate surgical excision of the cysts. During cardiac surgery, scolicidal solutions like iodine, ethanol, methylene blue, or hypertonic saline can be used to sterilize the cyst [10].

## Conclusion

Cardiac echinococcosis is a rare condition, posing a significant risk of rupture and thus carrying a potentially perilous course if left untreated. Its clinical presentation is varied, often with subtle or misleading symptoms. Non-invasive imaging modalities play a crucial role in its diagnosis, offering valuable insights into the underlying pathology.

MRI (Magnetic Resonance Imaging) provides detailed information regarding the precise anatomical location and characteristics of both external and internal structures affected by the disease. On the other hand, CT (Computed Tomography) imaging excels in highlighting wall calcifications, aiding in the identification of specific features associated with cardiac echinococcosis. Combining the strengths of these imaging techniques enhances diagnostic accuracy and informs treatment decisions, thereby improving patient’s outcome.

## References

[1] Yılmaz R , Akpınar YE, Bayramoglu Z, et al. Magnetic resonance imaging characteristics of cardiac hydatid cyst. Clin Imaging 2018;51:202–8. 29860193 10.1016/j.clinimag.2018.05.016

[2] Murphy T , Kean B, Venturini A, et al. Echinococcus cyst of the left ventricle: report of a case with review of the pertinent literature. J Thorac Cardiovasc Surg 1971;61:443–50. 5545589

[3] Yaliniz H , Tokcan A, Salih OK, et al. Surgical treatment of cardiac hydatid disease: a report of 7 cases. Tex Heart Inst J 2006;33:333–9. 17041691 PMC1592285

[4] Shojaei E , Yassin Z, Rezahosseini O. Cardiac hydatid cyst: a case report. Iran J Public Health 2016;45:1507–10. 28028503 PMC5182260

[5] Oraha AY , Faqe DA, Kadoura M, et al. Cardiac hydatid cysts; presentation and management. A case series. Ann Med Surg (Lond) 2018;30:18–21. 10.1016/j.amsu.2018.04.001. 29946454 PMC6016321

[6] Petik B , Hazirolan T, Uysal G, et al. Cardiac hydatid cysts: computed tomography and magnetic resonance imaging findings of the 5 cases. J Comput Assist Tomogr 2015;39:816–9. 10.1097/RCT.0000000000000284. 26196344

[7] Finn JP , Nael K, Deshpande V, et al. Cardiac MR imaging: state of the technology. Radiology 2006;241:338–54. 10.1148/radiol.2412041866. 17057063

[8] Mehra S , Garga UC. Left ventricular myocardial hydatid cyst. Appl Radiol 2018;36–8. 10.37549/AR2490.

[9] Tabesh H , Ahmadi Tafti H, Ameri S. Unusual presentation of interventricular hydatid cyst: a case report. Iran J Public Health 2015;44:130–3. 26060784 PMC4450000

[10] Shehatha J , Alward M, Saxena P, et al. Surgical management of cardiac hydatidosis. Tex Heart Inst J 2009;36:72–3. 19436793 PMC2676529

